# *FAS* promoter polymorphisms and serum sFas level are associated with increased risk of nerve damage in Bangladeshi patients with Guillain-Barré syndrome

**DOI:** 10.1371/journal.pone.0192703

**Published:** 2018-02-12

**Authors:** Zhahirul Islam, Israt Jahan, Rijwan U. Ahammad, Mohammad Shahnaij, Shamsun Nahar, Quazi D. Mohammad

**Affiliations:** 1 Laboratory Sciences and Services Division, International Centre for Diarrheal Disease Research (icddr,b), Dhaka, Bangladesh; 2 School of Medicine, Nagoya University, Nagoya, Japan; 3 National Institute of Neurosciences and Hospital, Dhaka, Bangladesh; Johns Hopkins University, UNITED STATES

## Abstract

Guillain-Barré syndrome (GBS) is an autoimmune disorder of the peripheral nervous system triggered by molecular mimicry between pathogen lipopolysaccharides and host nerve gangliosides. Polymorphisms in the Fas receptor (*FAS*) and Fas ligand (*FASL*) genes may potentially alter the elimination of autoreactive immune cells and affect disease susceptibility or disease severity in GBS. We detected single nucleotide polymorphisms (SNPs) in *FAS* (-1377G/A and -670A/G) and *FASL* (-843C/T) in a prospective cohort of 300 patients with GBS and 300 healthy controls from the Bangladeshi population. Genotype distributions were not significantly different between patients with GBS and healthy controls. The *FAS* -670 AG heterozygous (*P* = 0.0005, OR = 2.5, 95% CI = 1.5–4.2) and GG homozygous (*P* = 0.0048, OR = 2.6, 95% CI = 1.3–5.0) genotypes were more common in patients with anti-GM1 antibodies than patients without anti-GM1 antibodies. The *FAS* -670 G allele was more prevalent in anti-GM1 antibody-positive than -negative patients (*P* = 0.0002, OR = 1.9, 95% CI = 1.4–2.7) and also in patients with the axonal subtype than demyelinating subtype (*P* < 0.0001, OR = 4.8, 95% CI = 2.3–10.1). The 1377G/-670G GG haplotype was significantly associated with the axonal subtype (*P* < 0.0001) and anti-ganglioside antibody-positivity (*P* = 0.0008) in GBS. Serum sFas (237.5 pg/mL vs. 159.5 pg/mL; *P* < 0.0001) and sFasL (225.1 pg/mL vs. 183.4 pg/mL; *P* = 0.0069) were elevated in patients with GBS compared to healthy controls, and among patients with high serum sFas was associated with severe GBS (*P* = 0.0406). In conclusion, this study indicates *FAS-FASL* promoter SNPs may promote the production of cross-reactive anti-ganglioside antibodies in GBS.

## Introduction

Since global eradication of poliomyelitis [1], Guillain-Barré syndrome (GBS) has become the most frequently occurring post-infectious immune-mediated acute flaccid paralysis of the peripheral nervous system [2]. GBS is a heterogeneous disorder [3] that is mainly associated with precedent *Campylobacter jejuni* infection [4]. Molecular mimicry between *C*. *jejuni* lipooligosaccharides (LOS) and nerve gangliosides that elicits cross-reactive antibodies is the most widely accepted mechanism responsible for GBS, and is strongly associated with the axonal subtype (AMAN) of GBS [5,6]. Despite its established association with *C*. *jejuni*, post-infectious development of GBS occurs rarely after antecedent infection (in about 1 in 1000 cases) [7,8], indicating host factors are likely to influence susceptibility to GBS.

The Fas receptor (Fas)-Fas ligand (FasL) apoptotic pathway participates in elimination of autoreactive B and T cells involved in molecular mimicry to maintain immune homeostasis; hence, this pathway may protect against autoimmune diseases such as GBS [9,10]. Single nucleotide polymorphisms (SNPs) in the *FAS-FASL* promoter regions may alter gene expression and result in aberrant Fas-mediated apoptotic responses [11]; these mechanisms could potentially be involved in the pathogenesis or affect disease severity in GBS. Fas and FasL are membrane-bound apoptotic-signaling molecules that can trigger the cellular death signal cascade in response to cross-linkage of Fas and FasL [12]. Several studies have found an association between *FAS-FASL* polymorphisms and autoimmune diseases, including systemic lupus erythematosus (SLE), multiple sclerosis, primary Sjogren’s syndrome, and even GBS [12–15]. In fact the polymorphic *FAS* -670 G allele has been associated with a decreased risk of developing multiple sclerosis [13], but no significant association with GBS was evident [15].

Aberrant activation of the Fas-FasL pathway may also increase the levels of serum soluble Fas (sFas) and FasL (sFasL) in autoimmune pathologies [16]. The sFas receptor molecule, which is generated by alternative splicing and lacks the transmembrane segment, may contribute to disease pathogenesis by binding to FasL to prevent apoptosis in autoreactive or Fas-expressing lymphocytes [17]. On the other hand, sFasL is cleaved from membrane-bound FasL by matrix metalloproteinase (MMP), and possesses the ability to induce apoptosis by binding to FasL [18]. Increased levels of serum sFas and sFasL have been reported in SLE [19] and may indicate an aberrant immune response characterized by interference with Fas-FasL-mediated apoptosis [20].

Even though a Dutch group has extensively studied *FAS-FASL* polymorphisms, they could not establish *FAS-FASL* SNPs as a general susceptibility factor for GBS [15]. However, SNPs in the promoter regions of the genes encoding Fas and sFas were associated with the presence of anti-ganglioside antibodies in patients with GBS; this is the only report of an association between *FAS-FASL* polymorphisms and GBS to date [15]. Considering the lack of available data, further studies of populations from outside Europe may help to determine the relevance of *FAS-FASL* SNPs to host susceptibility and disease severity in GBS. In Bangladesh, most patients who develop GBS are severely affected and the disease has a high rate of mortality [21]; the clinical characteristics of patients with GBS are different to patients from other regions of the world [22]

Therefore, we determined the distribution of SNPs in the promoter regions of *FAS* (-1377G/A and -670A/G) and *FASL* (-843C/T) in patients with GBS and healthy controls from the Bangladeshi population. Serum sFas and sFasL levels were also quantified to examine the associations with the clinical and immunological features of GBS.

## Materials and methods

### Patients and healthy control individuals

A total of 300 Bangladeshi patients with GBS (206 males, 94 females) aged 4 to 60-years-old (median, 28-years-old) admitted to Dhaka Medical College Hospital (DMCH) between 2011 and 2013 participated in this prospective study. All patients fulfilled the National Institute of Neurological and Communicative Disorders and Stroke (NINDS) diagnostic criteria for GBS [23]. Clinical, electrophysiological and serological data were obtained for all enrolled patients. Pre-treatment blood samples were collected at entry (day of enrollment) before starting specific treatment e.g. IVIg or plasma exchange. Clinical data were assessed at standard time-points (2 weeks, 4 weeks, 6 months, 1 year after entry) to evaluate disease outcome.

Two-thirds of patients with GBS (225, 75%) had a preceding illness mostly diarrhea (110, 50%) or respiratory tract infection (50, 22%), and almost half of patients (144, 48%) had an anti-ganglioside antibody (GM1) response. Electrophysiological data indicated 62% of patients (120, acute motor axonal neuropathy [AMAN]; 9, acute motor and sensory axonal neuropathy [AMSAN]) had the axonal subtype of GBS and 23% (48) had the demyelinating subtype (acute inflammatory demyelinating polyneuropathy [AIDP]); electrophysiological tests were not performed for 93 (31%) patients. Disease severity was assessed using GBS disability scores (GDS); 73% (219) of patients had a GDS ≥ 3 and were classified as severely affected, 27% (81) had a GDS ≤ 2 and were classified as mildly affected [15].

A total of 300 healthy controls (144 females, 156 males) with no history of neurological or chronic medical illnesses (aged 17 to 75-years-old, median age, 34-years-old) who were genetically unrelated to the patients with GBS were recruited (Table 1). This study was approved by the ethics committees of both the icddr,b, and Dhaka Medical College and Hospital (DMCH); written informed consent was provided by all enrolled participants.

**Table 1:** Demographic, clinical and electrophysiological characteristics of the 300 patients with GBS and 300 healthy controls.

Characteristic		GBS patients n = 300 (%)	HC n = 300 (%)
**Sex**	Male/female (%)	206/94 (69/31)	156/144 (52/48)
**Age**	Median age, years (range)	28 (4–60)	34 (17–75)
**Antecedent events**		225 (75)	-
	Diarrhea	110 (49)	-
	Respiratory infection	50 (22)	-
	Other	65 (29)	-
Mean days between onset of weakness and study inclusion		11 days	-
**Severity based GBS disability scale scores (GDSs)**			
	Severely affected (GDS ≥ 3)	219 (73)	-
	Mildly affected (GDS ≤ 2)	81 (27)	-
**Anti-ganglioside antibodies**			
	GM1-positive	144 (48)	6 (2)
	GM1-negative	156 (52)	294 (98)
**Subtype of GBS (*n* = 207)**			
	AMAN	120 (58)	-
	AMSAN	9 (4)	-
	AIDP	48 (23)	-
	Unclassified	30 (15%)	-

AMAN, acute motor axonal neuropathy; AMSAN, acute motor-sensory axonal neuropathy; AIDP, acute inflammatory demyelinating polyneuropathy

### Genotyping of *FAS* and *FASL* SNPs

Blood samples for genomic DNA isolation were collected from the 300 patients with GBS and 300 healthy controls into lithium heparin-coated anti-coagulation blood collection tubes. The QIAamp^®^ DNA Blood Midi Kit (Qiagen, Hilden, Germany) was used to isolate genomic DNA according to the manufacturer’s instructions. The promoter regions containing the *FAS* -1377 G/A and -670 A/G and *FASL* -843 C/T polymorphisms were amplified in a single real-time PCR assay on a LightCycler capillaries (Roche Diagnostics, Mannheim, Germany) using previously described primers, probes, PCR master mix and thermocycling conditions [15]; melting curve profiling was performed after amplification. A color compensation set (Roche Molecular Biochemicals) was used to balance the *FAS-FASL* channels. A direct counting method was used to evaluate the haplotype frequency of *FAS* -1377 G/A and -670 A/G receptor polymorphisms.

### Determination of serum IgG against GM1 ganglioside

Serum samples separated from pretreatment blood were analyzed using enzyme-linked immunosorbent assay (ELISA) to determine the presence of the most commonly detected anti-ganglioside (GM1) IgG antibodies in patients with GBS in Bangladesh [5]. The mean difference in the optical densities (d-OD) of two GM1-coated wells and two uncoated wells was used to define anti-GM1 reactivity. A previously determined cut-off value of ≥ 0.20 for Bangladeshi patients was defined as IgG-positive serum [24].

### Quantification of soluble Fas and FasL in serum

Serum sFas and sFasL were measured in 94 patients with GBS (11 mildly affected and 83 severely affected) who had complete follow-up data (2 weeks, 4 weeks, 6 months and 1 year after entry) and 57 age- and sex-matched healthy control individuals. Commercially available sandwich ELISA kit (Diaclone Research, Besancon, France) was used to measure these proteins according to the manufacturer’s instructions [15].

### Statistical analysis

The genotype and allele frequencies of patients and healthy controls were compared using Fisher’s exact test for the *FAS* and *FASL* gene polymorphisms. Hardy-Weinberg equilibrium for the healthy control was confirmed using the Chi-square test. The non-parametric Mann–Whitney *U*-test was used to compare the levels of serum sFas and sFasL between patients and healthy controls. The associations between serum sFas and sFasL levels and each the *FAS/FASL* genotype for the studied SNPs in patients with GBS were analyzed using the Kruskal-Wallis test. The associations between the clinical features of GBS and genotypes in patients were analyzed using Fisher’s exact test. The Bonferroni adjustment was performed to exclude type I errors in multiple tests. Haplotype and allele frequencies were estimated by simple gene counting and data was processed using Microsoft Excel 2007. Statistical analyses were performed using GraphPad Prism (version 5; GraphPad Software) and SPSS (20.0 version; IBM) software. A probability level (*P*) of less than 0.05 was adopted as a significance criterion. Allele specific *P*-values and odds ratios (ORs) were estimated using Fisher’s exact test.

## Results

### Distribution of *FAS* and *FASL* SNPs among patients with GBS and healthy controls

The distribution of two SNPs in the *FAS* receptor gene in promoter region (-1377 G/A and -670 A/G) and one SNP in the *FASL* ligand gene in promoter region (-843 C/T) among patients with GBS and healthy controls are illustrated in Table 2.

**Table 2:** *FAS* and *FASL* gene polymorphisms in patients with GBS and healthy controls.

*FAS* polymorphism	GBS patients (*n* = 300)	HC (*n* = 300)	*P*-value	OR (95% CI)
*FAS* -1377 G/A				
GG	183	198	-	Reference
AG	105	93	0.26	1.2 (0.9–1.7)
AA	12	9	0.66	0.8 (0.3–1.9)
*FAS* -670 A/G				
AA	135	129	-	Reference
AG	114	126	0.42	0.9 (0.6–1.2)
GG	51	45	0.81	1.1 (0.7–1.7)
*FASL* -843 C/T				
TT	108	120	-	Reference
CT	129	123	0.41	1.2 (0.8–1.7)
CC	63	57	0.37	1.2 (0.8–1.9)

GBS, Gullain Barré syndrome; HC, healthy controls; OR, odds ratio; 95% CI, 95% confidence interval.

All three SNPs were in Hardy-Weinberg equilibrium in both patients with GBS and healthy controls. No significant differences in the genotype and allelic distributions of the *FAS* gene polymorphisms at -1377 and -670 were apparent between patients with GBS and healthy controls. However, the *FAS* -1377 AG heterozygous (35% vs. 31%; *P* = 0.26) and -670 GG homozygous variant (17% vs. 15%; *P* = 0.81) were more common in patients with GBS than healthy controls, but were not significantly associated with GBS. No significant association was found between genotype/allele distributions of the *FASL* (-843 C/T) polymorphism and GBS.

### Association of *FAS* and *FASL* SNPs with subgroups of patients with GBS based on clinical and laboratory data

We classified the patients with GBS based on clinical and serological features. The comparison of genotype and allele frequencies between anti-ganglioside antibody-positive and -negative patients is shown in Table 3.

**Table 3:** *FAS* and *FASL* promoter polymorphisms in patients with GBS stratified by clinical and immunological features.

**SNP**	**Anti-GM1-Ab response**			
	**Positive** **(*n* = 144)**	**Negative** **(*n* = 156)**	***P*-value**	**OR (95% CI)**
***FAS* -670 A/G**				
AA	48	87	-	Reference
AG	66	48	0.0005	2.5 (1.5–4.2)
GG	30	21	0.0048	2.6 (1.3–5.0)
A allele	162	222	-	Reference
G allele	126	90	0.0002	1.9 (1.4–2.7)
	**Electrophysiological classification**			
	**Axonal subtype** **(*n* = 129)**	**Demyelinating subtype** **(*n* = 48)**	***P*-value**	**OR (95% CI)**
***FAS* -670 A/G**				
AA	45	30	-	Reference
AG	63	12	0.0018	3.5 (1.6–7.6)
GG	21	6	0.1076	2.3 (0.8–6.4)
A allele	153	72	-	Reference
G allele	105	24	0.0064	2.1 (1.2–3.5)

Anti-GM1-Ab, anti-GM1 antibody; OR, odds ratio; 95% CI, 95% confidence interval; NC, not calculated; adjusted *P* value 0.0167.

We observed a significant association between the genotype distribution of the *FAS*-670 A/G polymorphism and anti-GM1 antibody-positivity. Both the heterozygous AG (*P* = 0.0005, OR = 2.5, 95% CI = 1.5–4.2) and homozygous GG (*P* = 0.0048, OR = 2.6, 95% CI = 1.3–5.0) variants were more frequent among anti-GM1 antibody-positive patients. The G allele at position -670 was also significantly more frequent among anti-GM1 antibody-positive patients (*P* = 0.0002, OR = 1.9, 95% CI = 1.4–2.7).

We also compared genotype and allele frequencies within the subgroups of GBS patients based on electrophysiological data (Table 3). The *FAS* -670 heterozygous AG variant (*P* = 0.0018, OR = 3.5, 95% CI = 1.6–7.6) and G allele (*P* = 0.0064, OR = 2.1, 95% CI = 1.2–3.5) were significantly more common among patients with the axonal subtype than the demyelinating subtype of GBS (Table 3). However, there were no significant associations between the *FAS* and *FASL* genotype and allele frequencies and the severity of GBS.

### Association between *FAS* -1377/-670 haplotypes and GBS

Four different haplotype combinations of the *FAS* -1377 G/A and -670 A/G promoter polymorphisms were observed: *FAS* -1377G; -670A (GA), -1377G; -670G (GG), -1377A; -670G (AG) and -1377A; -670A (AA). Haplotype frequency was not significantly different between the patients with GBS and healthy controls.

Subgroup analysis revealed the GG haplotype was significantly more frequent among anti-GM1 antibody-positive than anti-GM1 antibody-negative patients with GBS (*P* = 0.0008, OR = 1.9, 95% CI = 1.3–2.8). The GG haplotype was also more common in patients with the axonal subtype compared to the demyelinating subtype of GBS (*P* < 0.0001, OR = 4.8, 95% CI = 2.5–9.5; Table 4). No association was apparent between *FAS* haplotypes and severity of GBS.

**Table 4:** Associations between *FAS* -1377/-670 haplotypes and GBS.

Categories	*FAS* -1377/-670 haplotypes			
	GA	GG	AG	AA
Healthy controls	310	176	42	72
GBS patients	306	162	48	84
*P*-value	-	0.64	0.57	0.37
OR (95% CI)	Reference	0.9 (0.7–1.2)	1.2 (0.7–1.8)	1.2 (0.8–1.6)
Anti-GM1 Ab-positive GBS	129	99	27	33
Anti-GM1 Ab-negative GBS	174	69	21	48
*P*-value	-	0.0008	0.0864	0.801
OR (95% CI)	Reference	1.9 (1.3–2.8)	1.7 (0.9–3.2)	0.9 (0.6–1.5)
Axonal subtype of GBS	108	87	18	45
Demyelinating subtype of GBS	72	12	0	12
*P*-value	-	< 0.0001	NC	0.0107
OR (95% CI)	Reference	4.8 (2.5–9.5)	NC	2.5 (1.2–5.0)

OR, Odds ratio; 95% CI, 95% confidence interval; NC, Not calculated; Adjusted *P* value 0.0125.

### Serum levels of sFas and sFasL

**Fig 1:**
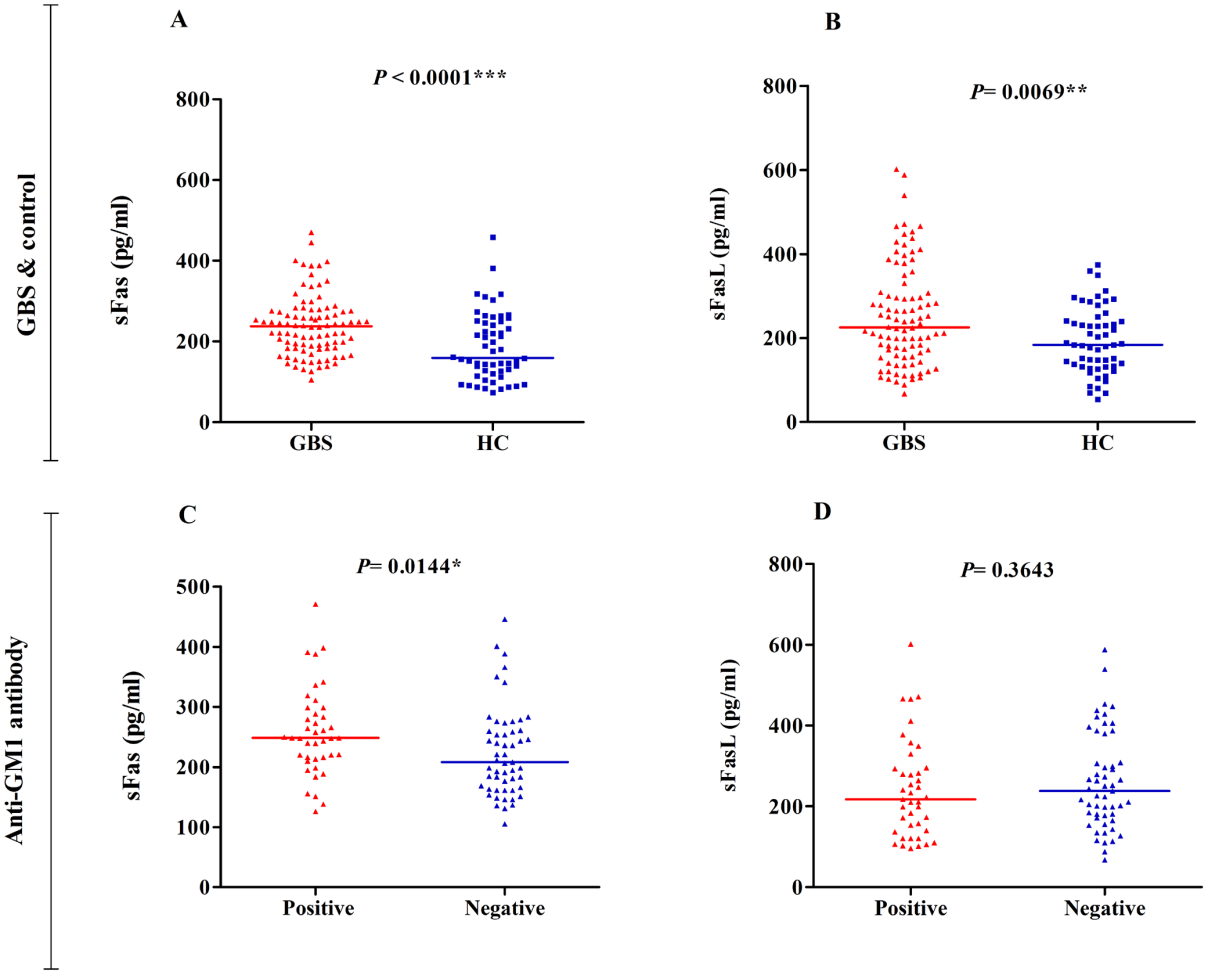
Serum levels of sFas & sFasL in patients with GBS and healthy controls and subgroups of patients with GBS. Serum levels of **(A)** sFas & **(B)** sFasL in patients with GBS (*n* = 94) and healthy controls (*n* = 57) and **(C)** sFas & **(D)** sFasL patients with GBS stratified by the presence and absence of anti-GM1 antibodies. The thick horizontal lines indicate median serum sFas and sFasL levels; *P < 0.05, Mann–Whitney *U*-test.

Serum sFas acts as an inhibitor of Fas receptor-ligand binding in extracellular spaces, which impairs homeostatic regulation of immune responses. Thus, we quantified circulating serum sFas and sFasL levels in the patients with GBS and healthy controls. The median serum levels of sFas (237.5 pg/mL vs. 159.5 pg/mL; *P* < 0.0001) and sFasL (225.1 pg/mL vs. 183.4 pg/mL; *P* = 0.0069) were significantly higher in patients with GBS than healthy controls (“Fig 1” and “S1 File”).

**Fig 2:**
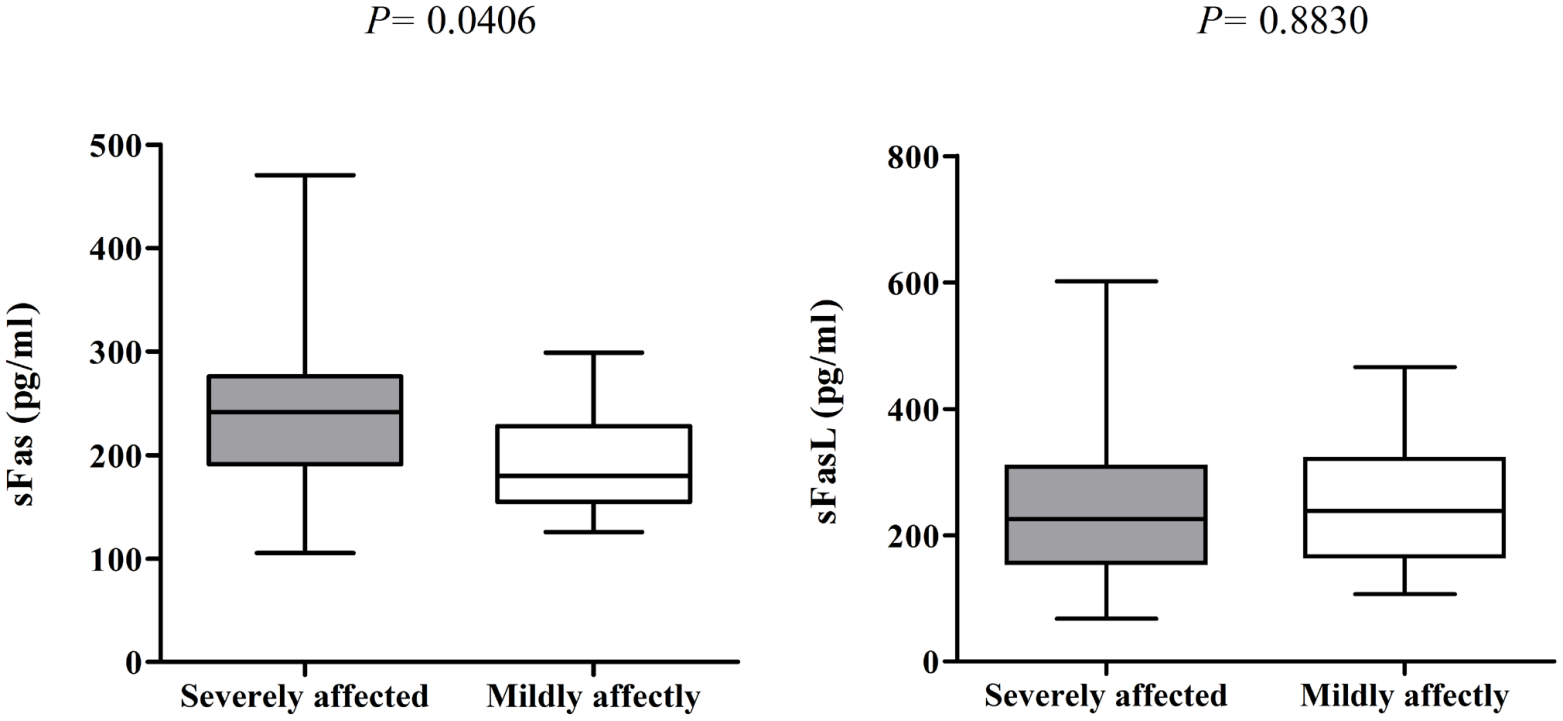
Serum levels of sFas and sFasL in severely and mildly affected patients with GBS. Severity of disease was assessed using the GBS disability score, a GDS ≥ 3 was defined as severely affected and ≤ 2, as mildly affected. The Whisker-box plots indicate the minimum, maximum and median values; *P < 0.05, Mann–Whitney *U*-test.

Among the patients with GBS, sFas level was significantly higher in anti-GM1 antibody-positive patients than patients without anti-GM1 antibodies (248.4 pg/mL vs. 208.2 pg/mL; *P* = 0.0144). In contrast, the sFasL level was higher in patients without anti-GM1 antibodies than anti-GM1 antibody-positive patients, though this trend was not significant (238.4 pg/mL vs. 217.4 pg/mL; *P* = 0.3643, “Fig 1”). The sFas level was significantly higher in severely affected patients compared to mildly affected patients (246.8 pg/mL vs. 180.1 pg/mL; *P* = 0.0406), but the sFasL level was not significantly different between mildly affected and severely affected patients (237.8 pg/mL vs. 223.3 pg/mL; *P* = 0.8830; “Fig 2”). No significant associations were observed between sFas and sFasL levels and any *FAS-FASL* genotype.

## Discussion

To the best of our knowledge, only one previous study has assessed the relationships between *FAS/FASL* promoter polymorphisms in patients with GBS [15]. Fas-FasL play a crucial role in elimination of inflammatory immune cells from the nervous system and are implicated in several neurodegenerative diseases [25–27], thus *FAS-FASL* genotypes may affect susceptibility and disease severity. In this study, we did not observe any significant association between the *FAS-FASL* promoter SNPs examined and susceptibility to GBS in the Bangladeshi population. However, *FAS* receptor polymorphisms, particularly the -670 A/G SNP, were significantly more common among anti-GM1 antibody-positive patients with GBS. In addition, patients with GBS had significantly elevated serum sFas and sFasL levels, though no associations were observed between sFas or sFasL levels and the various genotypes of the SNPs studied.

We studied the possibility that *FAS-FASL* genotypes may influence the clinical course of GBS. The AG and GG genotypes of the *FAS* -670 SNP were significantly associated with the presence of anti-GM1 antibodies in patients with GBS. This finding may be related to the increased risk of autoimmune reactions against nerve gangliosides (GM1) in patients with GBS. Though we performed the serological test against other nerve gangliosides (GD1a and GQ1b), but, we did not include these gangliosides in analysis for the low frequency of anti-ganglioside antibody-positivity. The *FAS* -670 G allele has been associated with reduced transcriptional activity and hence is hypothesized to be responsible for reduced expression of *FAS* [11]. We observed an association between the -670 G allele and anti-ganglioside antibody-positivity in patients with GBS, which indicates reduced expression of *FAS* leads to lower levels of apoptosis of autoreactive immune T cells and B cells. Reduced elimination of these immune cells may be responsible for the host ganglioside cross-reactivity that has been proposed to underlie the pathogenesis of GBS. Therefore, SNPs in the *FAS* receptor gene could be one of the major host susceptibility factors engaged in autoantibody production in GBS.

The *FAS* -670 AG genotype and G allele had extremely high frequencies in the axonal subtype of GBS compared to the demyelinating subtype. This could be explained by the fact the Fas-mediated apoptotic pathway not only eliminates activated T cells, but is also involved in axonal degeneration and regeneration. Nerve Schwann cells express high levels of the Fas receptor and FasL on the cell surface membrane and may induce Fas-FasL-mediated apoptosis of invading nerve cells [28]. However, elevated serum sFasL induces elimination of inflammatory cells and consequently promotes axonal regeneration [29]. Though the axonal subtype is more common among patients from the Asian continent [30], it would be useful to compare our findings with studies from other ethnic groups or populations in which the demyelinating subtype predominates.

Increased serum sFas and sFasL levels have been reported in several immune-mediated diseases [12,18]. In this study, sFas and sFasL were significantly elevated in the pretreatment serum samples of patients with GBS compared to healthy controls. Moreover, the subgroup of patients with anti-GM1 antibody-positivity had increased serum sFas levels compared to patients without anti-GM1 antibodies. Elevated serum sFas inhibits Fas-FasL-mediated apoptosis by interacting with FasL [31], and lead to the development of more severe forms of GBS. In contrast, higher sFasL levels in patients lacking anti-GM1 antibodies may promote apoptosis in Fas-expressing invading inflammatory or autoreactive immune cells [29]. However, the higher sFasL level in anti-GM1 antibody negative patients compared to antibody-positive patients was not statistically significant. Moreover, it was previously shown that healthy individuals carrying the *FAS* -670 AA genotype have significantly higher sFas levels [10]. This study could not establish an association between elevated serum levels of the sFas and sFasL proteins and the examined *FAS* and *FASL* genotypes.

Soluble FasL may induce axonal regeneration by eliminating invading cells [29] and may therefore promote recovery in patients with GBS. In agreement with this suggestion, we observed higher sFasL levels in mildly affected patients (GDS ≤ 2) than severely affected patients (GDS ≥ 3); however, we could not establish any significant association between sFasL levels and disease severity. However, we observed a significant elevation in sFas serum levels among severely affected patients compared to mildly affected patients, as previously reported in a study of a Dutch cohort [15]. The severity of GBS tends to be high in Bangladesh [22], thus we could not establish any significant association between elevated sFasL levels in the eleven mildly affected patients. This small sample size may have also limited our ability to detect a significant association.

In conclusion, this study indicates polymorphisms in the *FAS* and *FASL* genes may not affect genetic susceptibility to GBS in the Bangladeshi population. However, analysis of *FAS* polymorphisms in subgroups of patients with and without anti-GM1 antibodies suggests involvement of the *FAS* -670 AG genotype and *FAS* -1377/ -670 GG haplotype as stimulators of the cross-reactive immune response following antecedent infection in GBS. More extensive studies with larger sample sizes that include patients from different geographical regions are required to further clarify the immunopathogenic role of *FAS* SNPs in GBS.

## Supporting information

S1 File Serum level (row data file) of sFas and sFasL in patients with GBS and healthy controls. (XLS)
